# The Shared Use of Extended Phenotypes Increases the Fitness of Simulated Populations

**DOI:** 10.3389/fgene.2021.617915

**Published:** 2021-02-03

**Authors:** Guilherme F. de Araújo, Renan C. Moioli, Sandro J. de Souza

**Affiliations:** ^1^Bioinformatics Multidisciplinary Environment, Instituto Metrópole Digital, Universidade Federal do Rio Grande do Norte, Natal, Brazil; ^2^Brain Institute, Universidade Federal do Rio Grande do Norte, Natal, Brazil; ^3^Institutes for Systems Genetics, West China Hospital, University of Sichuan, Chengdu, China

**Keywords:** extended phenotypes, Moran process, simulated platform, system biology, network

## Abstract

Extended phenotypes are manifestations of genes that occur outside of the organism that possess those genes. In spite of their widespread occurrence, the role of extended phenotypes in evolutionary biology is still a matter of debate. Here, we explore the indirect effects of extended phenotypes, especially their shared use, in the fitness of simulated individuals and populations. A computer simulation platform was developed in which different populations were compared regarding their ability to produce, use, and share extended phenotypes. Our results show that populations that produce and share extended phenotypes outrun populations that only produce them. A specific parameter in the simulations, a bonus for sharing extended phenotypes among conspecifics, has a more significant impact in defining which population will prevail. All these findings strongly support the view, postulated by the extended fitness hypothesis (EFH) that extended phenotypes play a significant role at the population level and their shared use increases population fitness. Our simulation platform is available at https://github.com/guilherme-araujo/gsop-dist.

## Introduction

The main idea behind the extended phenotype (Dawkins, 1982) lies in how far a gene effect can reach. According to Dawkins (1982), a gene can have its effect outside of the physical body of the bearer with several types of consequences, including environmental ones. In that way, a gene extends its effect in, for example, a beaver’s dam, a spider’s web, or a bird’s nest. Although the examples above represent physical structures, extended phenotypes are also seen as signals (Schaedelin and Taborsky, 2009), social interactions (Wang et al., 2008), or manipulations of behaviors (Hoover et al., 2011). Extended phenotypes are described in all taxonomic kingdoms, from viruses (Hoover et al., 2011) to humans (Dixson, 2019). Although the widespread existence of extended phenotypes is clearly established in contemporaneous evolutionary biology (reviewed in Bailey, 2012), the degree and intensity of its effects are still controversial (Hunter, 2009; Bailey, 2012).

Besides the obvious effect of the extended phenotypes in the fitness of the organism who generated it, many authors have discussed their indirect genetic effects (Laland, 2004; Wang et al., 2008; de Souza, 2013; Fisher et al., 2019). One type of indirect genetic effect is through social interactions mediated by extended phenotypes (Wang et al., 2008). Extended phenotypes could also affect other parties’ fitness through niche construction, as discussed by Laland (2004). A few years ago, the extended fitness hypothesis (EFH) had been proposed, which states that extended phenotypes serve as a link between individual and group selection (de Souza, 2013). Suppose the following scenario: a spider web is abandoned by the individual who built it. A different spider from the same species can then use that web, which in turn contributes to the fitness of the new owner. Remarkably, the spider web silk can vary within the same species depending on environmental factors, and protein-deprived spiders produce silk that is more efficient at capturing preys than that produced by protein-fed members of the same species (conspecifics; Blamires et al., 2017, 2018), resulting in “silk performance landscapes across nutrient space” (Blamires et al., 2016). In another example, a bird’s nest shape impact on its thermal profile, which in turn, has been shown to influence offspring fitness (Olson et al., 2006; Martin et al., 2017). Thus, using an extended phenotype built by others may have greater advantage than simply reducing the costs associated to building the phenotype. However, the fitness effects of such biological plasticity mechanisms and their impact on individual and group selection are not fully understood.

The basis of the EFH is the fact that individuals can use extended phenotypes built by conspecifics. Thus, extended phenotypes possess indirect genetic effects in individuals who can use them. Group selection emerges naturally as a consequence of such shared use of extended phenotypes by members of the same species/group. As discussed by de Souza (2013), there are several examples of the use of available extended phenotypes by conspecifics, including cases with spiders (Schuck-Paim and Alonso, 2001), cichlids (Schaedelin and Taborsky, 2009), and wasps (Brockmann et al., 1979). For instance, a beaver’s dam may cause a significant environmental change that goes beyond the immediate ecosystem (Gurnell, 1998; Rosell et al., 2005). More recently, Fisher et al. (2019) showed that food hoards, identified as extended phenotypes, built by red squirrels outlived the individuals who built them and were subsequently used by conspecifics. More interestingly, different features of the food hoards, like size, affected the fitness of the subsequent owner. While the data from Fisher et al. (2019) fit predictions made by EFH, such empirical models are hard to find and study. One alternative is the use of computer simulations to either compare distinct evolutionary scenarios or to study the role of a given parameter, in this case extended phenotypes, in the evolutionary process.

This led us to develop a computer simulation framework to test some premises of the EFH. Here, we show that extended phenotypes can, *per se*, increase the fitness of individuals who produce them. More importantly, however, populations that produce and share extended phenotypes outrun populations that only produce them. A mathematical treatment allowed us to derive variables that can be evaluated regarding their role in the fitness of the tested populations. A bonus linked to the shared use of extended phenotypes is strongly associated with winning populations in our simulations. All these findings support the view that the shared use of extended phenotypes is an important contributor to selection at population level. We made our simulation platform available at https://github.com/guilherme-araujo/gsop-dist.

## Materials and Methods

### Simulation Steps

The simulation protocol consists of three steps: graph generation, main simulation, and plot/analysis. At the graph generation step, the type of graph, the number of nodes, and the density of the graph are defined (Steger and Wormwald, 1999). The generated graph is then read by the main simulation program, which accepts parameters related to the bonuses, maximum number of cycles, and number of samples and states in which each node type can transition into. Finally, the output is processed and the plots generated using the scripts available at the corresponding folder of the public repository of this simulation platform. All simulations were run in the High-Performance Computing Unit of the Federal University of Rio Grande do Norte, consisting of 64 blade computational nodes, each with two 16-core Intel Xeon E5-2698v3 processors and 128GB DDR4 RAM.

### Graph Generation

Graphs were generated using the *newtworkx* (Hagberg et al., 2008) package for the Python programming language. All graphs in the simulation described in this paper were generated with the *barabasi_albert_graph* function of this package, with parameters *n* = 500 and *m* = 4.

### Main Simulation

**Figure 1:**
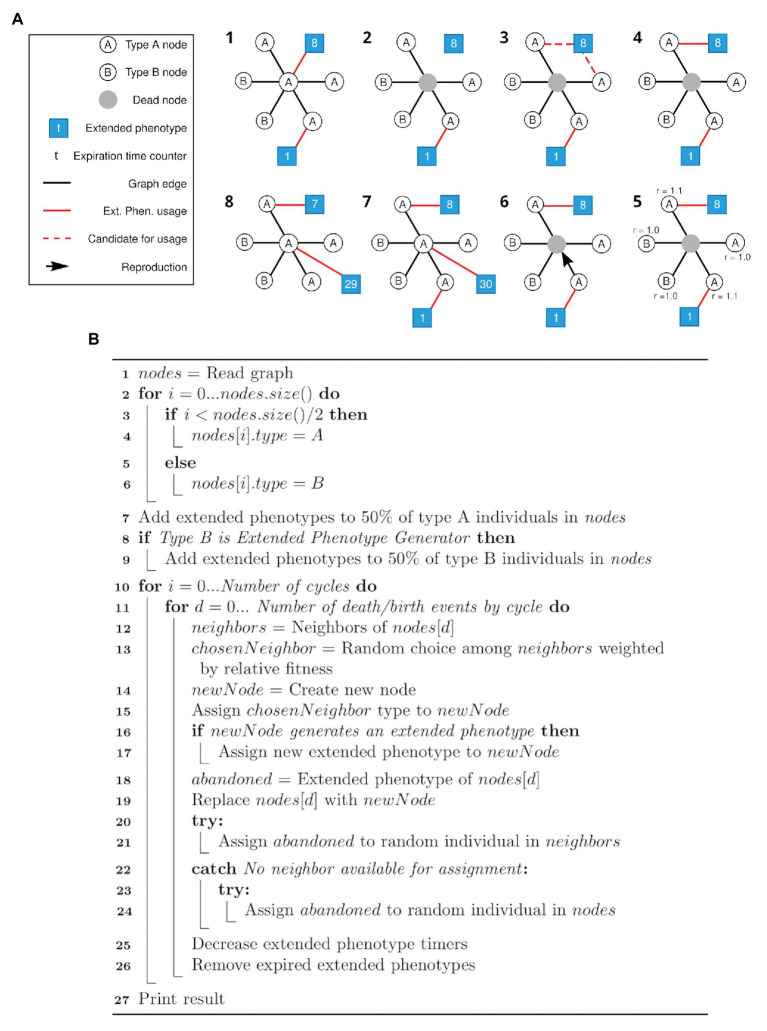
Schematic view of the simulation framework and respective pseudocode. **(A)** All steps (1–8) of a cycle of the framework are depicted. After the initial setup of the network (1), random individuals are selected to die (like the gray node in 2). Its associated extended phenotype becomes available (3) and one of the neighbors of the same type (in this case, type A) and without an associated extended phenotype is selected to gain the available extended phenotype (4). Selection of a node to duplicate and occupy the position of the dead node is based on a weight matrix (5, 6), as described in the text. A new node has a chance to generate an extended phenotype attached to itself (7). Each extended phenotype has an expiration time (*t*) represented by the number in the respective squares (7, 8). Step 8 represents the step 1 of the new cycle. For clarity, only the central node is represented with all its connections. **(B)** Pseudocode for the simulation framework described above **(A)**.

**Figure 2:**
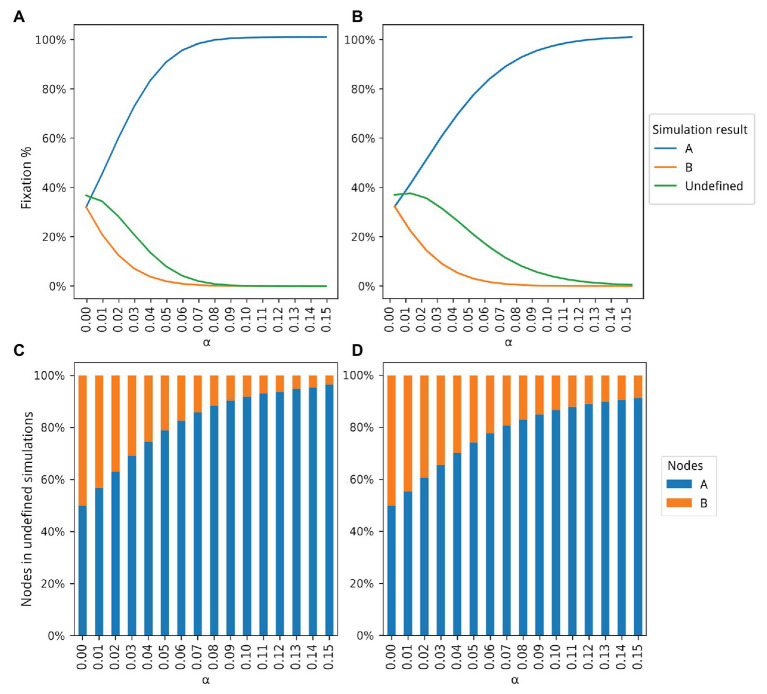
Dynamics of populations A and B according to 5 million simulations for different values of *α*. The blue and orange lines in **(A,B)** show how many simulations ended with the fixation of types A and B, respectively. The green line in **(A,B)** shows how many simulations ended without the fixation of either type, that is, undefined simulations. Proportions of type A and B individuals in the undefined simulations are shown in **(C,D)**. **(A)** Only population A is able to produce and share extended phenotypes. **(B)** Both populations can produce extended phenotypes but only population A is able to share extended phenotypes. **(C)** Proportions of type A and B individuals for the simulations represented by the green curve shown in **(A)**. **(D)** Proportion of type A and B individuals for the simulations represented by the green curve shown in **(B)**.

**Figure 3:**
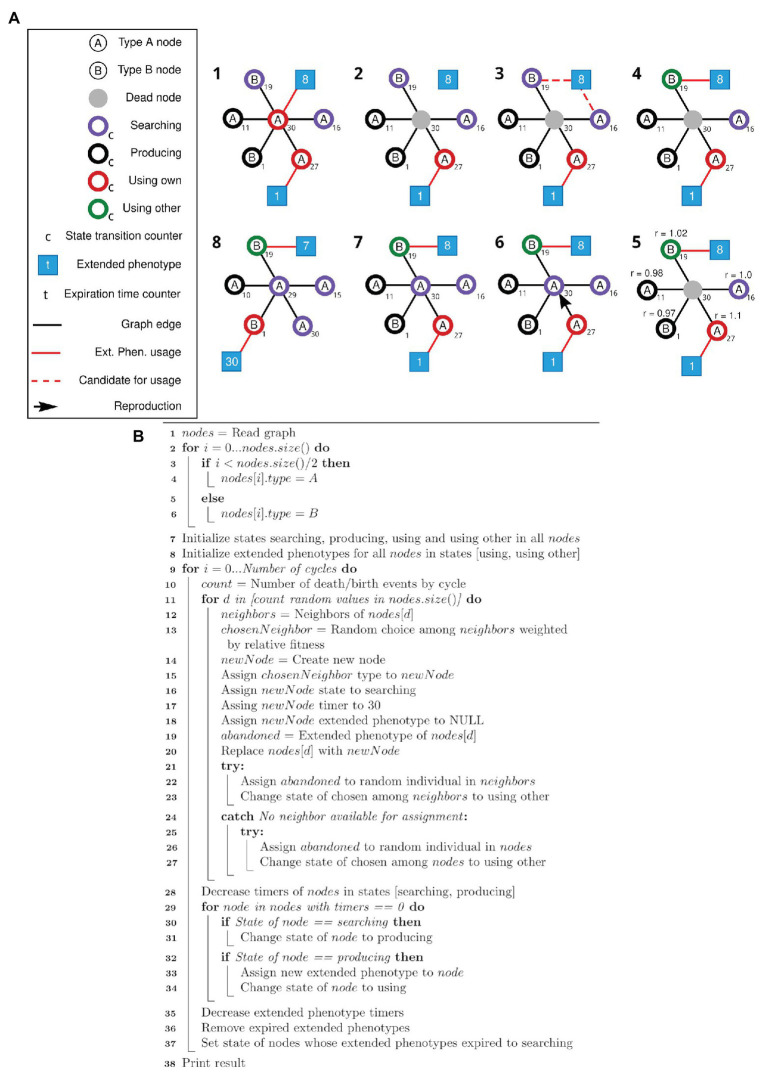
Schematic view of the modified simulation framework and respective pseudocode. **(A)** Nodes of types A and B can search, produce, and use its own or use other extended phenotypes. After the initial setup of the network (1), random individuals are selected to die (2). The associated extended phenotype becomes available, and one of the neighboring nodes in the Searching state is selected to gain the available extended phenotype (3, 4). Selection of a node to duplicate and occupy the position of the dead node is based on a weight matrix (5, 6), according to the state of each node. Node state transition and expiration time counters (*t*) are updated, and states and extended phenotypes are adjusted accordingly (7, 8). Step 8 represents step 1 of the new cycle. **(B)** Pseudocode for the simulation framework described above **(A)**.

**Figure 4:**
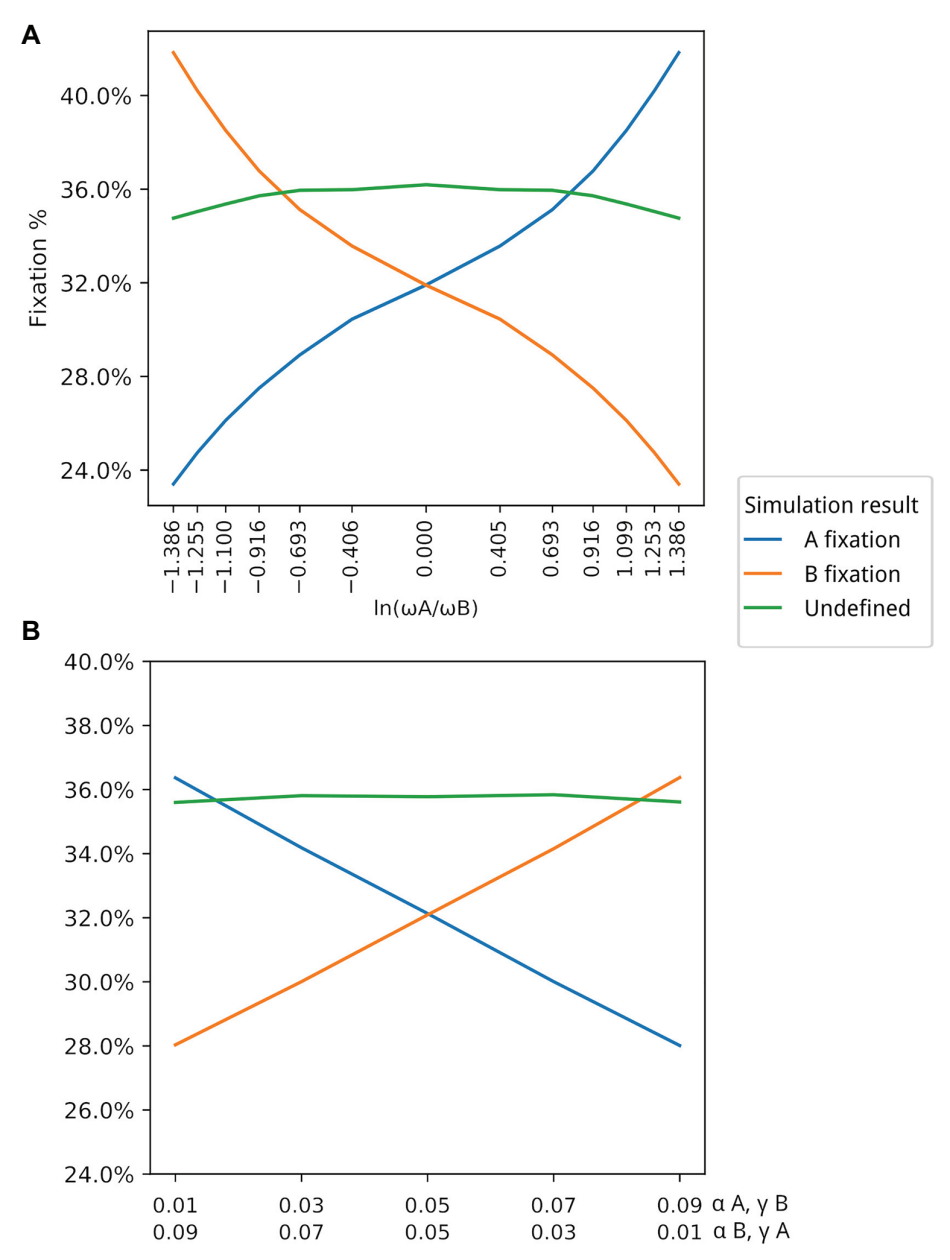
Association between *ω* and winning populations. *Y* axis in both graphs represents the average fixation % in the corresponding simulations. **(A)** Association between *ω* and winning populations (those with higher fixation rate). For this simulation, β=0.03 and γ=0.02 for both populations. **(B)** In this simulation, both *α* and *γ* are changed under the restriction that $\omega_{A}=\omega_{B}$ Populations with γ>α are winners in situations where $\omega_{A}=\omega_{B}.$ Values in the first line in the *X* axis correspond to $\alpha_{A}$ and $\gamma_{B}$. Values in the second line of the X axis correspond to $\alpha_{B}$ and $\gamma_{A}$.

The algorithm for the first simulation is described as a pseudocode in Figure 1B. The first simulation implements the framework described in Figure 1A, and generates data for plotting Figure 2. The algorithm for the second simulation is described as a pseudocode in Figure 3B. The second simulation implements the framework described in Figure 3A, and generates data for plotting Figure 4.

For the first algorithm, two sets of data were generated, the first for the simulation where B individuals do not generate any extended phenotype, and the second where both types generate extended phenotypes, but only type A individuals can reuse them. These two sets of data resulted in the plots seen in Figure 2. The second algorithm was used to generate another two sets of data, which resulted in the data seen in Figure 4.

In the first pseudocode (Figure 3B), the nodes are first (line 1) load from the graph generated in the first simulation step, described in “Graph generation.” In lines 2–6, nodes are initialized with type A or B. In lines 7–9, the extended phenotypes are initialized, and 50% of all individuals start with an extended phenotype. The verification at line 8 is related to the difference between the simulations that generated Figures 2A,B, since in the first set of data type B individuals do not generate extended phenotypes. Next, for each cycle (line 10) at each death/birth event (line 11), a random neighbor of the dying individual is chosen weighted according to its relative fitness (lines 12–13). The new individual is created having the same type of the chosen neighbor (lines 14–15), and if it belongs to a type which generates extended phenotypes – only A in the first, and both types in the second simulation – it occupies the extended phenotype (lines 16–17). If the dying individual had an extended phenotype, an attempt is made to assign it to one of the other neighbors or a random individual in the population (lines 18 and 20–24). The dying individual is then replaced by the new one (line 19) and finally the extended phenotype timers are decreased and those who expired are removed (lines 25–26).

The second algorithm starts like the previous one (lines 1–6), then initializes the states according to parameters defined by the user (lines 7–8). Then, for each death/birth event at each cycle (lines 9–11), a random neighbor of the dying individual is chosen weighted according to its relative fitness (lines 12–13) and a new node is created, always at the searching state, with no extended phenotype attached (lines 14–18). If the dying individual had an extended phenotype, an attempt is made to assign it to one of the other neighbors or a random individual in the population (lines 19 and 21–27). This individual will be transitioned to the “using other” state (lines 23 and 27). The dying individual is then replaced by the new one (line 19). Finally, all states and extended phenotype time counters are updated, its states transitioned and expired extended phenotypes removed (lines 28–37).

All simulations are provided only with the random graphs generated in the previous step and the parameters described in section “Simulation parameters.” For each of the four sets of data described previously, 1,000 graphs were generated, and 5,000 samples were generated with each of the 1,000 graphs, resulting in 5,000,000 total samples for each x-axis data point of each set of data.

The sets of data for the first and second simulation, which generated data for Figure 2, had 15 subsets of data each, varying the *α* value for A from 0.0 to 0.15 in both simulations, and for B in the second simulation. In the first simulation, the *α* value of B is fixed at 0.0. Every subset consists of 5,000,000 samples, generated in the previously described way, with the intention of removing any influence a particular characteristic of a randomly generated graph could have had on the final result.

The third set of data, which resulted in Figure 4A, varied the values of *α* and *γ* for A, in order to change the values of *ω* for the population of type A individuals, such that *ω_A_* divided by *ω_B_* varies from 0.25 to 4, resulting in the plotted log values seen on the *x*-axis of Figure 4A. The 13 subsets of data generated each point in the *x*-axis scale of this figure.

The fourth set of data resulted in Figure 4B. The *α* and *γ* values for A and B were set between 0.01 and 0.09, as seen on the label of the *x*-axis of Figure 4B in all five subsets of data.

### Plot and Analysis

The plots were generated using the data sets previously described. Figures 2A–D, were generated from the data produced by the first and second simulations, respectively, both based on the first simulation framework. Figures 4A,B depict the data generated from the latter data sets, produced by the simulation configured according to the second simulation framework. All plots were generated using the *matplotlib* (Hunter, 2007) package for the Python programming language.

### Simulation Parameters

Parameters for each simulation and the scripts used to generate them – those who resulted in Figures 2, 4 – are available at the public repository of this simulation platform. Below there is a brief description of each parameter from the main simulation program.

1. Samples – number of full simulations to be run with the currently loaded graph. The used value was 5,000 for all simulations.
2. Cycles – Simulation cycle limit. The simulation is considered undefined if it ends without fixation of either type A or B.
3. *α* values for types A and B.
4. *β* values for types A and B. If set to −1, this node type will not transition into “producing” state. The values are set to −1 in the simulations based on the first framework.
5. *γ* values for types A and B. If set to −1, this node type will not reuse abandoned extended phenotypes. This is the case for type B individuals on the sets of simulations based on the first framework.
6. Percentage of nodes at each state at the beginning of the simulation. For the simulations based on the framework described by Figure 1, only “with” or “without” extended phenotype states are available. This is achieved by setting percentages for “producing” and “using other” to zero.
7. Extended phenotype time. After generation, an extended phenotype will last a number of cycles before it expires. This time counter continues even after the extended phenotype is reused. If it has, for example, 10 cycles remaining when its original occupier dies, it will still have 10 cycles left whether it is reused or not. All simulations in this work had this parameter set to 30 cycles.
8. State time. For the simulations based on the framework described by Figure 3, states “searching” and “producing” last for a certain number of cycles before transitioning. The other two states, “using” and “using shared,” depend on the extended phenotype time. All simulations based on the second framework had it set to 30 cycles.
9. Extended phenotype birth generation chance. This parameter is relevant for simulations based on the framework described by Figure 1. It defines the chance of a new node having an extended phenotype attached to it, and was set to 50% on those simulations. On simulations based on the framework described by Figure 3, it is set to zero, since in these simulations, the extended phenotypes are generated by nodes transitioning from the “producing” state, instead of at birth.

See Supplementary Material for a more detailed description of the values passed to each parameter at each simulation set.

## Results

### Simulation Framework

An established approach for modeling the evolution of populations is the Moran Process (Moran, 1958). It is a simple stochastic model used to describe finite populations and can be used to simulate events, such as mutation and genetic drift by describing the probabilistic dynamics in a population containing two alleles, one of which can ultimately dominate the population. More recently, random scale-free graphs have been used to adapt the Moran Process to a more friendly simulation framework (Lieberman et al., 2005). These graphs share many characteristics of naturally occurring populations, such as in natural and artificial networks of relationships (Barabási and Albert, 1999). Therefore, it is suitable for modeling a generic population providing the conditions to test the EFH.

Thus, a population of individuals was modeled under the Barabási-Albert network model. This model generates a random graph that follows a power-law distribution of node degree, favoring the formation of clusters of highly connected nodes. The network grows according to preferential attachment, where new edges are more likely to be linked to nodes with higher degrees. In the original Moran Process adapted by Lieberman et al. (2005), all nodes begin with the same status. An individual of a different status is introduced into this population and by neutral drift or selection all other individuals can become bearers of the second status. This is achieved by a death-birth process, where an individual is randomly chosen to die, and in its place, a new individual is born. This individual is chosen based on a probability matrix calculated according to the neighbors of the dead node, weighted by their relative fitness, which translates into a numerical value representing its ability to reproduce. Regular nodes have relative fitness *r* = 1, and the “mutant” individuals have a relative fitness *r* = 1 + *α*, where *α* is the bonus/penalty provided by the mutation.

Here, a similar model was used to evaluate the effect of the shared use of extended phenotypes in the fitness of a population (see Figure 1A for a schematic view of our simulation framework). We started by generating a network with 500 nodes (individuals) with a parameter *m* = 4, which is the minimum number of edges for any given node. One modification of the Moran process implemented in the present model is that nodes are classified either as a type A (250 nodes) or B (250 nodes) since the start of the simulation. Our framework was designed to compare two populations composed of either type A or type B individuals. Here, the death-birth process was adapted for the extended fitness context by taking into consideration the production and use of extended phenotypes. For each set of parameters, we run 5 million simulations (first, 1,000 random Barabási-Albert networks were designed and then for each one of them 5,000 simulations were run). A pseudocode for this first algorithm is presented as Figure 1B and detailed in Methods (section Main simulation).

### Extended Phenotypes Increase the Fitness of Populations

In our first experiment, type A individuals were modeled as individuals who can produce and use their own extended phenotypes, and reuse extended phenotypes left behind by dead conspecifics. Type B individuals do not produce or use extended phenotypes. The initial setup for all executed simulations comprised a start ratio of 1:1 for type A and B individuals and a renewal rate of 4%, where at each generation 4% of all nodes are selected to die, and new nodes are placed in their locations in the graph according to the probability matrix explained in the previous section. The *α* value represents the bonus, the adaptive advantage of the extended phenotype, and was set between 0.0 and 0.15 (0.01 step) for each batch of simulations.

As illustrated in Figure 1, individuals of type A start with a 50% probability of having an extended phenotype already attached. Only individuals with attached extended phenotypes are given the bonus value in their relative fitness. Newborn individuals of type A have also a 50% chance of generating new extended phenotypes attached to themselves. When type A individuals leave behind an extended phenotype after death, this can be occupied by one of their type A neighbors chosen at random with equal chance, as long as it is not already occupying an extended phenotype. If there is no neighbor of type A or all of them already have their own extended phenotype, a random individual of type A with an unattached extended phenotype is chosen anywhere in the graph, in case such an individual exists. Otherwise, the extended phenotype vanishes.

Figure 2A shows the results for all 5 million simulations for each bonus value (see Supplementary Material for details). With *α* = 0, both populations reach fixation at the same rate, as expected, with a higher number of simulations undefined. A simulation is classified as undefined when no fixation of either node type is achieved. As *α* increases, a higher number of fixations of type A occurs until almost the totality of experiments ends with the fixation of type A individuals. A plateau, close to the upper limit of 5 million simulations, is reached around *α* = 0.08. The number of undefined simulations also decreases, and it is also possible to observe that even in those simulations, there is a larger number of type A individuals as the bonus increases (Figure 2C). For example, at *α* = 0.04, 75% of all undefined simulations had a higher proportion of type A individuals.

### The Reuse of Extended Phenotypes Increases the Fitness of Populations

While the data in Figure 2A show that production and use of extended phenotypes increase the fitness of populations (See also Supplementary Figure 2), predictions of the EFH remained untested, namely that selection would favor groups where extended phenotypes are shared between conspecifics. Some of the simulation parameters were thus modified to perform such tests. Now, both types produce extended phenotypes but only type A individuals are able to reuse a given extended phenotype when it becomes available.

As before, individuals of types A and B start with a 50% probability of having an extended phenotype already attached, and newborn individuals of both types also have a chance of 50% of generating new extended phenotypes attached to themselves. The major difference between individuals of type A and B happens at death: type A individuals can leave behind an existing extended phenotype, which can be preferentially occupied by one of their type A neighbors as described in the previous simulation. On the other hand, the death of type B individuals causes the vanishing of the corresponding associated extended phenotypes and no reuse ever happens in this case. To eliminate the saturation effect, the extended phenotype half-life is the same for both individual types.

Figure 2B shows the results with this second proposed simulation. The number of simulations ending with the fixation of A still grows with rising *α* values, but at a slower pace, given that now type B individuals also produce and benefit from extended phenotypes. However, the observed advantage of type A individuals is still dramatic, even when both types generate extended phenotypes with the same bonus values. The major difference between the two simulation sets seems to be the number of undefined simulations, which is slightly higher in the second set of simulations (Figure 2B), where both populations produce extended phenotypes but only type A individuals are able to share them. For example, with *α* = 0.05 only 10% of all simulations in Figure 2A are classified as undefined while the same number is 25% in Figure 2B. Like in Figure 2C, the proportion of type A individuals in the undefined simulations shows a positive association with *α* (Figure 2D). The occupancy rate of type A individuals with extended phenotypes also increases, since now the ones abandoned by dead individuals can be occupied by them. This effect can be seen in more detail in Supplementary Figure 2.

### The Bonus Gained for Sharing Extended Phenotypes Has a Higher Impact in the Fitness of the Population

The previous simulations only considered a fitness bonus (*α*) for individuals that occupy an extended phenotype. This first, simple simulation can be enhanced to include parameters that reflect broader effects of extended phenotypes in both individual and group selection: (i) the benefit of using an extended phenotype built by yourself (*α*); (ii) the cost of building an extended phenotype (*β*), and (iii) the benefit of using an extended phenotype built by another individual (*γ*). One could think of a cost for searching for an extended phenotype previously built by someone else and now available, but there is no evidence that such behavior exists, and these encounters seem fortuitous. Based on the above, it is reasonable to think that the shared use of extended phenotypes will be favored when:

$$\frac{\gamma \;}{\alpha -\beta }>1$$ [1]

However, when comparing two populations (A and B in our simulations), a more appropriate equation is:

$$\omega_{i}=\left(\alpha_{i}-\beta_{i}\right)+\gamma_{i}$$ [2]

where $\omega_{i}$ is the absolute fitness of population A or B. Although selection parameters in equation [2] are described from the perspective of the individual, they are considered here at the population level. They represent average effects across the whole population. In that sense, after defining $\omega_{i},$ one could estimate the abundance of a given phenotype using an equation like 3:

$$n\left(g+1\right)=\omega_{i}n\left(g\right)$$ [3]

where *n*(*g*) is the abundance of the phenotype in generation *g*. For the sake of simplicity, we will focus on equation 2 for the remaining simulations.

A new simulation (schematically viewed in Figure 3A) was modeled to test equation [2]. The four behaviors previously described were translated into four states: “searching” (searching for an extended phenotype), “producing” (producing an extended phenotype), “using own” (using your own extended phenotype), and “using other” (using an existing extended phenotype built by someone else). Each of these states has a different associated relative fitness value at each simulation step. The “searching” state describes the default behavior. The individual is neither using nor producing an extended phenotype and if by chance, it encounters an unused one, it will occupy it. This state is the baseline behavior, with relative fitness *r* = 1. If the individual stays in the “searching” state for a specific amount of time (a specific number of cycles in the simulation – see Methods), it will transition into a “producing” state, meaning it searched for some time for an unoccupied extended phenotype and now started producing its own. Its relative fitness will be negatively affected by a *β* modifier since that individual will be spending time and resources building its own extended phenotype. After a certain amount of time, a producing node will transition into a “using own” state. It receives an *α* bonus for occupying an extended phenotype, as in previous simulations. After its extended phenotype time expires, the individual returns to a “searching” state. The “using other” state is set to individuals who, similarly to the previous simulations, are using extended phenotypes abandoned by dead individuals. The individuals in the “using other” state will receive a *γ* bonus. A pseudocode for this second algorithm is presented as Figure 3B and detailed in Methods (section Main simulation).

We first had to evaluate whether simulation data fit equation (2). By varying both *ω*_A_ and ω_B_ in different simulations (by changing the corresponding *α* bonus for each population while keeping *β* and *γ* fixed), we observed that there is indeed a strong positive association between *ω* and the winning population (the population with higher fixation rate), as can be seen in Figure 4A. Although there is a strong association between the value of *ω* and the winning population, there are also winning populations when *ω* for both populations have the same value ($\omega_{A}/\omega_{B}=1)$. This suggests that other parameters may have an impact on the simulation output. Thus, we decided to test the effect of each variable in the outcome of the simulations by exploring different values for each variable but always keeping $\omega_{A}=\omega_{B}$. This allowed us to evaluate the impact of each individual parameter, especially *α* and *γ*. Table 1 shows all parameter values for each set of simulations. Our data show that *β* does not seem to affect the outcome of the simulations in terms of proportions of A and B (Supplementary Figure 1). This is probably due to the fact that the value of *β* is the same for both populations. On the other hand, the values of *α* and *γ* are critical in defining which population dominates the simulation. In all simulated scenarios, the population with a higher *γ* wins, as shown in Figure 4B, suggesting that the fitness gained for using an existing extended phenotype has a more significant impact than the fitness for using your own extended phenotype.

**Table 1:** Values of *ω* for different values of *α*, *β,* and *γ.*

α_A_, *γ_B_*; α_B_, *γ_A_*	*β* = 0.01	*β* = 0.03	*β* = 0.05	*β* = 0.07	*β* = 0.09
0.01; 0.08	0.08	0.06	0.04	0.02	0.00
0.02; 0.07	0.08	0.06	0.04	0.02	0.00
0.03; 0.06	0.08	0.06	0.04	0.02	0.00
0.04; 0.05	0.08	0.06	0.04	0.02	0.00
0.05; 0.04	0.08	0.06	0.04	0.02	0.00
0.06; 0.03	0.08	0.06	0.04	0.02	0.00
0.07; 0.02	0.08	0.06	0.04	0.02	0.00
0.08; 0.01	0.08	0.06	0.04	0.02	0.00

## Discussion

Extended phenotypes have received significant interest since the original concept emerged in the early 80’s (Dawkins, 1982), especially their indirect effects in other individuals or environments (Dawkins, 2004; Bailey, 2012; de Souza, 2013; Blamires et al., 2018; Fisher et al., 2019). Research in the field has been limited by the paucity of empirical models in which extended phenotypes can be manipulated and different evolutionary models be compared. We have, therefore, generated a computer simulation platform to evaluate the effects of the production and shared use of extended phenotypes on the fitness of simulated populations. We were particularly interested in testing the EFH as proposed by de Souza (2013).

The platform is flexible and can be easily adapted to study different real biosystems. For example, population interaction is structured with graphs, whose topology can be reconfigured to accommodate different ecological networks. Also, evolutionary dynamics can be manipulated by changing the probabilities of encounter, interaction, production and reuse of extended phenotypes, and the bonus/penalty associated with each behavior. This flexible architecture can thus be used to study, formulate, and test hypotheses in diverse areas, from plant-soil-microbial communities (Terhorst and Zee, 2016) to cancer evolution (Ewald and Swain Ewald, 2013). In fact, the extended phenotype hypothesis has been linked to a myriad of phenomena and has recently sparked interest (Hunter, 2018), partly due to novel computer simulations and data processing techniques. In this way, we believe that our work, more than testing aspects of the EFH, expands the toolbox to unveil evolutionary dynamics.

Nevertheless, there are several issues regarding extended phenotypes that could be explored using our simulation platform. Extended phenotype plasticity and its effect on the fitness of individuals and populations (Blamires, 2010; Bailey, 2012; Katz et al., 2017; Blamires et al., 2018) is an example that could be explored in our computational framework. Furthermore, the interplay between extended phenotype plasticity and other features, like for example dietary conditions, as observed by Blamires et al. (2018) and Katz et al. (2017) could likewise be studied in the computational setup presented here.

We show that the shared use of extended phenotypes has a significant contribution to the absolute fitness of a given population. This gives support to the EFH. One interesting aspect of the EFH is the fact that it does not advocate mutually exclusive fundamental evolutionary processes. As discussed by de Souza (2013), the effect of EFH at the group level is a natural consequence of the shared use of extended phenotypes by conspecifics. Furthermore, this shared use of extended phenotypes does not involve cooperation since the two parties likely never met, as discussed by Fisher et al. (2019). The mathematical treatment provided here, although simple, allowed us to evaluate quantitatively the influence of different parameters in the fitness of the respective populations. In all scenarios tested, the shared use of extended phenotypes (quantified by the parameter *γ*) had a stronger influence on the fitness of the respective populations.

It is important to emphasize the assumptions and limitations of the strategy used in this report. There are, of course, intrinsic limitations derived from the simulated nature of the data. The different types of extended phenotypes (ranging from different physical structures to behaviors) bring also some challenges for an approach based on computational simulations. For example, the type of network used here (the Barabasi-Albert graph) may be more appropriate for some types of extended phenotype (like a web or a nest), while a regular network (where all nodes have the same degree) may be more appropriate for the study of the effect of a biofilm on the fitness of a bacterial population. Furthermore, few assumptions made in our simulations have the potential to affect our conclusions. First, no cost for searching for an existing extended phenotype was set in our simulations. This is a reasonable assumption since, to our knowledge, no such behavior has been described so far, and it is likely that the encounters are fortuitous. Furthermore, we have not taken into consideration the emergence of cheaters in our system (i.e., genetic variants that stop producing their own extended phenotypes and only use extended phenotypes of other individuals), which could also affect the evolutionary dynamics of the corresponding population. de Souza (2013) has discussed this issue but a formal evaluation through computer simulations needs to be done. Another interesting possibility, not explored here, is the modification of an existing extended phenotype by the individual who occupied it. These issues should be explored in the future.

## Data Availability Statement

Publicly available datasets were analyzed in this study. This data can be found at: https://github.com/guilherme-araujo/gsop-dist.

## Author Contributions

SS conceived the presented idea and wrote the first version of the manuscript. GA wrote all the scripts of the simulation platform and carried out the simulations. All authors contributed to the article and approved the submitted version.

### Conflict of Interest

The authors declare that the research was conducted in the absence of any commercial or financial relationships that could be construed as a potential conflict of interest.

## References

[ref1] Bailey N. W. (2012). Evolutionary models of extended phenotypes. Trends Ecol. Evol. 27, 561–569. 10.1016/j.tree.2012.05.011, PMID: 22832012

[ref2] Barabási A. -L. Albert R. (1999). Emergence of scaling in random networks. Science 286, 509–512. 10.1126/science.286.5439.509, PMID: 10521342

[ref3] Blamires S. J. (2010). Plasticity in extended phenotypes: orb web architectural responses to variations in prey parameters. J. Exp. Biol. 213, 3207–3212. 10.1242/jeb.045583, PMID: 20802123

[ref4] Blamires S. J. Blackledge T. A. Tso I. M. (2017). Physicochemical property variation in spider silk: ecology, evolution, and synthetic production. Annu. Rev. Entomol. 62, 443–460. 10.1146/annurev-ento-031616-035615, PMID: 27959639

[ref5] Blamires S. J. Martens P. J. Kasumovic M. M. (2018). Fitness consequences of plasticity in an extended phenotype. J. Exp. Biol. 221:jeb167288. 10.1242/jeb.167288, PMID: 29361580

[ref6] Blamires S. J. Tseng Y. H. Wu C. L. Toft S. Raubenheimer D. Tso I. M. (2016). Spider web and silk performance landscapes across nutrient space. Sci. Rep. 6:26383. 10.1038/srep26383, PMID: 27216252 PMC4877650

[ref7] Brockmann H. J. Grafen A. Dawkins R. (1979). Evolutionary stable nesting strategy in a digger wasp. J. Theor. Biol. 77, 473–496. 10.1016/0022-5193(79)90021-3, PMID: 491692

[ref8] Dawkins R. (1982). The extended phenotype: The long reach of the gene. Oxford: Oxford University Press.

[ref800] Dawkins R. (2004). Extended phenotypes - but not too extended. A reply to Laland, Turner and Jablonka. Biol. Philos. 19, 377–396. 10.1023/B:BIPH.0000036180.14904.96, PMID: 19255576

[ref9] de Souza S. J. (2013). “Extended fitness” hypothesis: a link between individual and group selection. Genet. Mol. Res. 12, 4625–4629. 10.4238/2013.October.17.5, PMID: 24222238

[ref10] Dixson B. J. W. (2019). Sexual selection and extended phenotypes in humans. Adapt. Hum. Behav. Physiol. 5, 103–107. 10.1007/s40750-018-0106-3

[ref11] Ewald P. W. Swain Ewald H. A. (2013). Toward a general evolutionary theory of oncogenesis. Evol. Appl. 6, 70–81. 10.1111/eva.12023, PMID: 23396676 PMC3567472

[ref12] Fisher D. N. Haines J. A. Boutin S. Dantzer B. Lane J. E. Coltman D. W.. (2019). Indirect effects on fitness between individuals that have never met via an extended phenotype. Ecol. Lett. 22, 697–706. 10.1111/ele.13230, PMID: 30740839

[ref13] Gurnell A. M. (1998). The hydrogeomorphological e•ects of beaver dam-building activity. Prog. Phys. Geo. Earth Environ. 22, 167–189. 10.1177/030913339802200202

[ref14] Hagberg A. Schult A. Swart P. (2008). “Exploring network structure, dynamics, and function using NetworkX”. in *Proceedings of the 7th Python in Science Conference*; August 19-24, 2008; 11–15.

[ref15] Hoover K. Grove M. Gardner M. Hughes D. P. McNeil J. Slavicek J. (2011). A gene for an extended phenotype. Science 333:1401. 10.1126/science.1209199, PMID: 21903803

[ref16] Hunter P. (2009). Extended phenotype redux. How far can the reach of genes extend in manipulating the environment of an organism? EMBO Rep. 10, 212–215. 10.1038/embor.2009.18, PMID: 19255576 PMC2658563

[ref17] Hunter P. (2018). The revival of the extended phenotype. EMBO Rep. 19:e46477. 10.15252/embr.201846477, PMID: 29871873 PMC6030696

[ref18] Hunter J. D. (2007). Matplotlib: A 2D graphics environment. Comput. Sci. Eng. 9, 90–95. 10.1109/MCSE.2007.55

[ref19] Katz N. Shavit R. Pruitt J. N. Scharf I. (2017). Group dynamics and relocation decisions of a trap-building predator are differentially affected by biotic and abiotic factors. Curr. Zool. 63, 647–655. 10.1093/cz/zow120, PMID: 29492026 PMC5804212

[ref20] Laland K. (2004). Extending the extended phenotypes. Biol. Philos. 19, 313–325. 10.1023/B:BIPH.0000036113.38737.d8

[ref21] Lieberman E. Hauert C. Nowak M. A. (2005). Evolutionary dynamics on graphs. Nature 433, 312–316. 10.1038/nature03204, PMID: 15662424

[ref22] Martin T. E. Boyce A. J. Fierro-Calderón K. Mitchell A. E. Armstad C. E. Mouton J. C. (2017). Enclosed nests may provide greater thermal than nest predation benefits compared with open nests across latitudes. Funct. Ecol. 31, 1231–1240. 10.1111/1365-2435.12819

[ref23] Moran P. A. P. (1958). Random processes in genetics. Proc. Camb. Phil. Soc. 54, 60–71.

[ref700] Olson R. C. Vleck C. M. Vleck D. (2006). Periodic cooling of bird eggs reduces embryonic growth efficiency. Physiol. Biochem. Zool. 79, 927–936. 10.1086/506003, PMID: 16927239

[ref25] Rosell F. Bozsér O. Collen P. Parker H. (2005). Ecological impact of beavers Castor fiber and Castor canadensis and their ability to modify ecosystems. Mammal Rev. 35, 248–276. 10.1111/j.1365-2907.2005.00067.x

[ref26] Schaedelin F. C. Taborsky M. (2009). Extended phenotypes as signals. Biol. Rev. 84, 293–313. 10.1111/j.1469-185X.2008.00075.x, PMID: 19382933

[ref27] Schuck-Paim C. Alonso W. J. (2001). Deciding where to settle: conspecific attraction and web-site selection in the orb-web spider *Nephilengys cruentata*. Anim. Behav. 62, 1007–1012. 10.1006/anbe.2001.1841

[ref28] Steger A. Wormwald N. (1999). Generating random regular graphs quickly. Probab. Comput. 8, 377–396. 10.1017/S0963548399003867

[ref29] Terhorst C. P. Zee P. C. (2016). Eco-evolutionary dynamics in plant-soil feedbacks. Funct. Ecol. 30, 1062–1072. 10.1111/1365-2435.12671

[ref30] Wang J. Ross K. G. Keller L. (2008). Genome-wide expression patterns and the genetic architecture of a fundamental social trait. PLoS Genet. 4:e1000127. 10.1371/journal.pgen.1000127, PMID: 18636101 PMC2442221

