# Supplementary material for: The Shared Use of Extended Phenotypes Increases the Fitness of Simulated Populations
Source: Front Genet. 2021 Feb 3;12:617915. doi: 10.3389/fgene.2021.617915 (PMC7886806; doi:10.3389/fgene.2021.617915)
Supplement: Supplementary file 1 [file Data_Sheet_1.docx]

Supplementary Figures

**
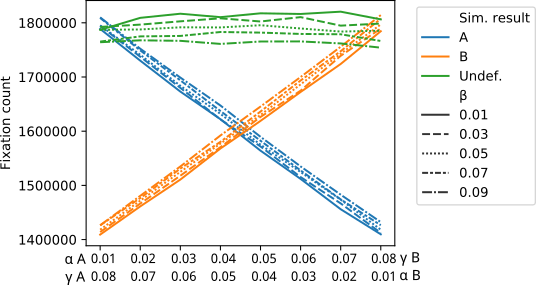
**

**Supplementary Figure 1.** Simulation results for different values of β. Different β values have little effect on the outcome of simulations using the second simulation framework.


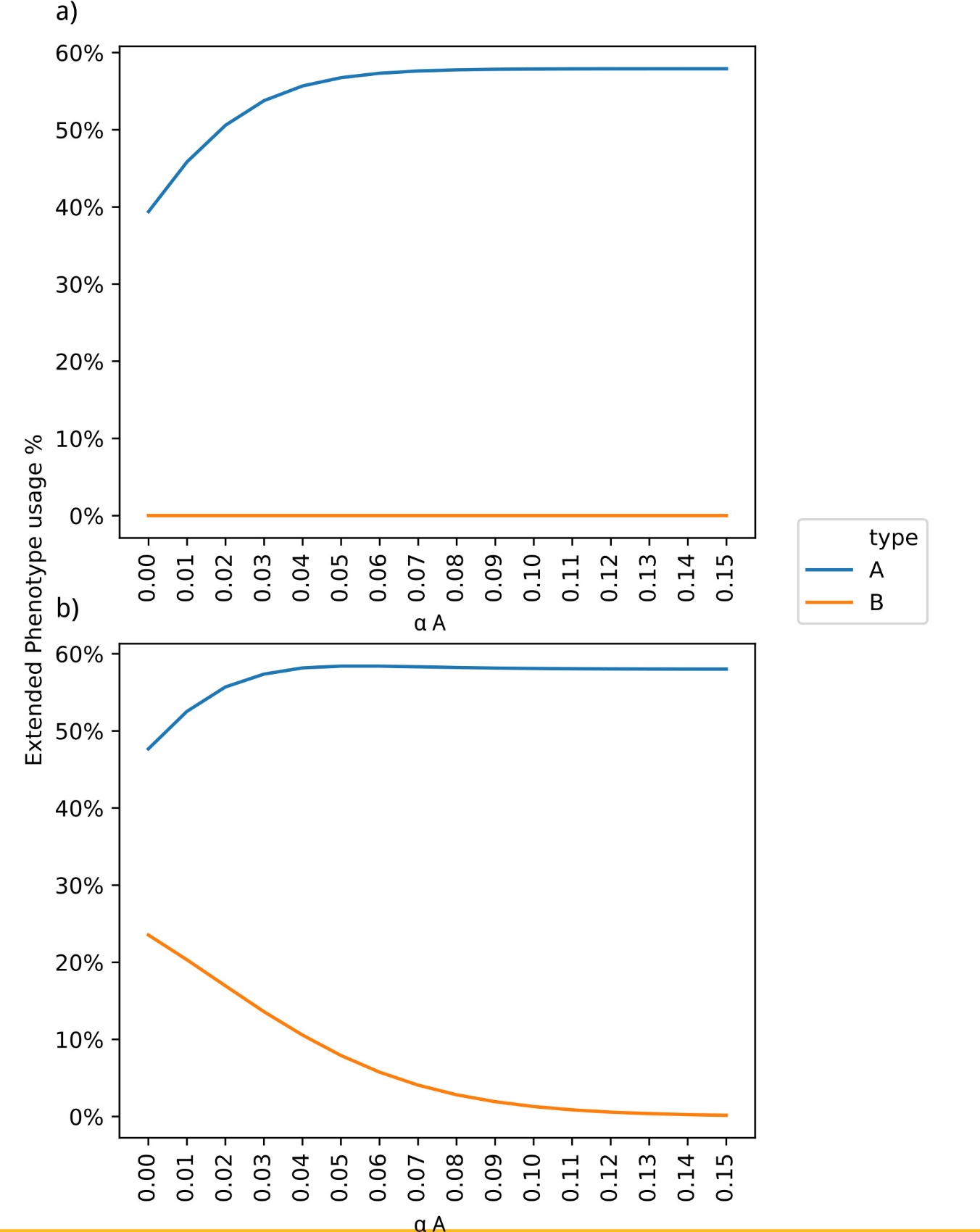


**Supplementary Figure 2** Percentage of individuals occupying extended phenotypes on the simulations based on the first simulation framework. Plots a) and b) were plotted using the same data which originated plots in figures 2a and 2b. On plot b) it is noticeable that even though the bonus of using an extended phenotype is zero, the occupancy rate of type A individuals is higher because of the reuse of the extended phyenotypes.
