# Supplementary material for: The Shared Use of Extended Phenotypes Increases the Fitness of Simulated Populations
Source: Front Genet. 2021 Feb 3;12:617915. doi: 10.3389/fgene.2021.617915 (PMC7886806; doi:10.3389/fgene.2021.617915)
Supplement: Supplementary file 2 [file Data_Sheet_2.docx]

**Supplementary Methods**

Below is a more detailed description of all parameters passed to the simulations, and how they generated the plots seen in figures 2 and 4.

1. For the first simulation set, which resulted in figures 2a and 2c

The code which generated the 16 simulation sub-sets which resulted in each x-axis value in figure 2a and 2c are avaliable at <https://github.com/guilherme-araujo/gsop-dist/tree/master/article/figure-2a-2c>. Each simulation is run with the following parameters, as described in the Methods section of the main article:

1. Samples 5,000
2. Cycles: 5,000
3. α: Varies from 0.00 to 0.15 for the A individuals. It is 0.00 in all simulation sets for type B.
4. β: -1, since this prevents individuals from transitioning into the building state. This only occurs in the last two simulations.
5. 𝛾: -1, since this prevents individuals from reusing extended phenotypes.
6. Percentage of nodes at each state in the beginning of the simulation: 50% with and 50% without extended phenotypes for type A individuals. 100% without extended phenotypes for type B individuals.
7. Extended phenotype time: 30 cycles expiration time.
8. State time: Not applicable to this simulation.
9. Extended phenotype birth generation chance: 50% for type A individuals. 0% for type B individuals.

These parameters are defined at each subfolder of the given link, at the exp.sh script.

After running the scripts at the sub-folders, the output files generated by them are read by the python scripts that generate figures 2a and 2c.

For figure 2a: <https://github.com/guilherme-araujo/gsop-dist/blob/master/article/figure-2a-2c/plot-05-25-1.py>

For figure 2c: <https://github.com/guilherme-araujo/gsop-dist/blob/master/article/figure-2a-2c/barplot.py>

1. For the second simulation set, which resulted in figures 2b and 2d

The code which generated the 16 simulation sub-sets which resulted in each x-axis values in figure 2b and 2d are avaliable at <https://github.com/guilherme-araujo/gsop-dist/tree/master/article/figure-2b-2d>. Each simulation is run with the following parameters, as described in the Methods section of the main article:

1. Samples: 5,000
2. Cycles: 5,000
3. α: Varies from 0.00 to 0.15 for the A and B individuals.
4. β: -1, since this prevents individuals from transitioning into the building state. This only occurs in the last two simulations.
5. 𝛾: -1 for B individuals, since this prevents individuals from reusing extended phenotypes. For A individuals, it is the same value given for α.
6. Percentage of nodes at each state in the beginning of the simulation: 50% with and 50% without extended phenotypes for both type A and type B individuals.
7. Extended phenotype time: 30 cycles expiration time.
8. State time: Not applicable to this simulation.
9. Extended phenotype birth generation chance: 50% for both type A and type B individuals.

These parameters are defined at each subfolder of the given link, at the exp.sh script.

After running the scripts at the sub-folders, the output files generated by them are read by the python scripts that generate figures 2b and 2d.

For figure 2b:

<https://github.com/guilherme-araujo/gsop-dist/blob/master/article/figure-2b-2d/plot-05-25-1.py>

For figure 2d:

<https://github.com/guilherme-araujo/gsop-dist/blob/master/article/figure-2b-2d/barplot.py>

1. For the third simulation set, which resulted in figure 4a

The code which generated the 13 simulation sub-sets which resulted in each x-axis value in figure 4a are avaliable at <https://github.com/guilherme-araujo/gsop-dist/tree/master/article/figure-4a>. Each simulation is run with the following parameters, as described in the Methods section of the main article:

1. Samples: 5,000
2. Cycles: 5,000
3. α: Varies from 0.02 to 0.08 for the A individuals. It is 0.02 in all simulation sets for type B.
4. β: 0.02 for all simulations, in both type A and type B individuals.
5. 𝛾: Varies from 0.02 to 0.08 in simulations where the α is fixed at 0.02, resulting in ω_A_/ ω_B_ varying from 0.25 to 4.
6. Percentage of nodes at each state in the beginning of the simulation: 40% Searching, 40% building, 10% using and 10% using other for both type A and type B individuals.
7. Extended phenotype time: 30 cycles expiration time.
8. State time: 30 cycles until individuals in the searching state transition into producing, and 30 cycles until producing individuals generate an extended phenotype and transition into using state.
9. Extended phenotype birth generation chance: 0% for both type A and type B individuals.

These parameters are defined at each subfolder of the given link, at the exp.sh script.

After running the scripts at the sub-folders, the output files generated by them are read by the python scripts that generate figure figure 4a: <https://github.com/guilherme-araujo/gsop-dist/blob/master/article/figure-4a/plot-05-25-wa-wb.py>

1. For the fourth simulation set, which resulted in figure 4b

The code which generated the 5 simulation sub-sets which resulted in each x-axis value in figure 4b are avaliable at <https://github.com/guilherme-araujo/gsop-dist/tree/master/article/figure-4b>. Each simulation in run with the following parameters, as described in the Methods section of the main article:

1. Samples: 5,000
2. Cycles: 5,000
3. α: Varies from 0.01 to 0.09 for the A individuals, and the inverse for B individuals.
4. β: 0.05 for all simulations, in both type A and type B individuals.
5. 𝛾: Varies from 0.01 to 0.09 for type A and B individuals, set at the inverse α value for each type.
6. Percentage of nodes at each state in the beginning of the simulation: 40% Searching, 40% building, 10% using and 10% using other for both type A and type B individuals.
7. Extended phenotype time: 30 cycles expiration time.
8. State time: 30 cycles until individuals in the searching state transition into producing, and 30 cycles until producing individuals generate an extended phenotype and transition into using state.
9. Extended phenotype birth generation chance: 0% for both type A and type B individuals.

These parameters are defined at each subfolder of the given link, at the exp.sh script.

After running the scripts at the sub-folders, the output files generated by them are read by the python scripts that generate figure figure 4b:

<https://github.com/guilherme-araujo/gsop-dist/blob/master/article/figure-4b/lineplot-4b.py>
